# Supplementary material for: Performance of immunological assays for universal and differential diagnosis of HTLV-1/2 infection in candidates for blood donations from the Brazilian Amazon
Source: PLoS One. 2024 Jul 5;19(7):e0298710. doi: 10.1371/journal.pone.0298710 (PMC11226060; doi:10.1371/journal.pone.0298710)
Supplement: S1 File — (DOCX) [file pone.0298710.s001.docx]

**Supplementary Material 1.** Immunological assays methods

**- Chemiluminescence assay (CLIA)**

The chemiluminescence assay (CLIA) is carried out in the HEMOAM Foundation’s serology sector as a screening step to identify donors that are positive for the virus. The procedure consists of a two-step immunoassay for the qualitative detection of antibodies to HTLV-1 and HTLV-2 in the serum, using the Alinity’s rHTLV-I/II kit (Abbott^®^), and is performed on the Alinity’s i-SCM 02 Ai01767 (Abbott^®^) equipment with a closed system. The sample, the HTLV-1 and HTLV-2 coated paramagnetic microparticles and the assay diluent are combined and incubated. Antibodies to HTLV-1 and HTLV-2 present in the sample bind to synthetic HTLV-1 and HTLV-2 peptides and microparticles coated with recombinant HTLV-2 antigen. The mixture is then washed. Synthetic HTLV-1 and HTLV-2 peptides and acridinium-labeled HTLV-1 recombinant antigen conjugate are added to create a reaction mixture and incubated. After a wash cycle, the pre-start and start solutions are added. The resulting chemiluminescent reaction is measured as relative light units (RLUs). There is a direct relationship between the number of antibodies to HTLV-1 and HTLV-2 in the sample and the RLUs detected by the system’s optics. The presence or absence of antibodies to HTLV-1 and HTLV-2 in the sample is determined by comparing the chemiluminescent RLUs in the reaction to the cutoff RLUs determined from an active calibration. According to the criteria of the Alinity’s rHTLV-I/II kit, a sample with RLUs >1.0 is considered reactive for HTLV-1/2 antibodies.

**- Western blot assay**

The western blot (WB) assay was performed with the HTLV blot 2.4 kit (MP Diagnostics^®^), following the guidelines of the manufacturer of the kit. The WB is used to define antigens derived from HTLV-1 and HTLV-2 proteins and is used as a confirmatory step that is capable of detecting and distinguishing between types 1 and 2 of the virus, as recommended by the Food and Drug Administration (FDA) and Brazilian Ministry of Health. The antigens used are recombinant proteins or synthetic peptides purified and fixed on a nylon membrane. The antigenicity displayed by these proteins and peptides is common to HTLV-1 and HTLV-2 antibodies or specific to one of the two types of the virus and allows confirmation and discrimination of these in the analyzed sample. Two gag bands (p19 I/II, p24 I/II) and two env bands (gp46 I/II, gp21 I/II) are applied as antigens without type specificity, and are used to confirm the presence of antibodies against HTLV- 1/2. The type antigens specified for HTLV-1 (gag p19-I, env gp46-I) and HTLV-2 (env gp46-II) are applied to differentiate between HTLV-1 and HTLV-2 infections. The sample to be tested can be plasma or serum from collections with tubes containing anticoagulant EDTA, citrate or heparin. Samples must be previously centrifuged for cell separation, and to carry out the test you will need a strip from the kit impregnated with HTLV-1/ 2 antigens, which will be placed in a trough and submerged in 1 mL of diluent solution containing 10 µL of homogenized sample. Make sure that the trough is sufficiently large so as to not spill the diluent solution/sample. The strip must be completely submerged in the solution and have the lines of the membrane facing upwards; cover the cavities with an adhesive sealant. The samples are then incubated, placing the support on a shaker or oscillator, and shaken overnight (16 ± 2 h) at room temperature (18 – 25 °C). The adhesive sealants should then be carefully removed to avoid cross-contamination, the strips should be washed 3 times (5 minutes) with 1 mL of washing solution from the kit. Subsequently, 1 mL of conjugate solution, provided in the kit, is added to each test well, incubating the plate with the conjugate and placing the test support on the shaker or oscillator for 30 minutes at room temperature (18 – 25 °C). Then, the strips should be washed 3 times (5 minutes) with 1 mL of washing solution (from the kit). 1 mL of substrate solution (from the kit) is added to each test well, incubating the plate with the substrate and placing the support on the shaker or oscillator, and shaking for 30 minutes at room temperature (18 – 25 °C). At the end, the liquid must be aspirated and 1 mL of stopping solution (from the kit) added to each well, followed by incubation with the stopping solution, placing the support on the shaker or oscillator, and shaking for 10 - 30 minutes at room temperature (18 – 25 °C). Subsequently, the strips must be removed from the test wells and, using tweezers, placed with the membrane side facing upwards on absorbent paper. When the strips are completely dry, you can interpret the results. To speed up the drying process, place the strips in an oven at 37 °C for 30 minutes or use a dryer for 1 minute. Processed strips maintain their color as long as they are stored in a dark place. When the sample does not contain HTLV-specific antibodies, only a slight background color develops and the control lines that were impregnated into the membrane to check the quality of the test appear.

**- Flow cytometry assay**

Antigenic support for immunofluorescence by flow cytometry was created with MT-2 and MoT cells kept in an oven at 37 ºC, 5% CO2, 95% humidity with medium changes and weekly passages. In summary, the cells are seeded in 75 cm² tissue culture flasks, with RPMI-20% fetal bovine serum (FBS) medium and maintained in an oven at 37 ºC, 5% CO_2_, 95% humidity for 72 h to promote growth of the cells. After this time, the cell suspension in log growth phase will be homogenized and centrifuged at 1,200 rpm for 10 min to eliminate clumps of cells. Non-aggregated cells will be washed with PBS supplemented with 0.5% FBS by centrifugation at 1,200 rpm for 10 min in 50 mL conical tubes. The cell suspension will be fixed by resuspension in a fixative solution (10.0 g/L of paraformaldehyde, 10.2 g/L of sodium cacodylate and 6.65 g/L of sodium chloride pH 7.2). The cell suspension will be adjusted to 1.0 x 10^6^/mL in PBS-0.5% FBS. Differential labeling of cell lines: based on previous work carried out by the Integrated Biomarker Research Group, differential fluorescent labeling is performed with the fluorochrome Alexa Fluor 647 with different concentrations, with concentrations of 0.002 µg/mL for MT-2 and 0.04 µg/mL for MoT, which are defined as the best for differentiating one cell line from another in terms of mean fluorescence intensity (MFI). The methodologies differ in the use of the cell line, with the MT-2 used for the FC-Simplex, typing step, and the MoT and MT-2 lineage used in the FC-Duplex to perform viral typing. Searching for IgG1 antibodies using flow cytometry: To search for anti-HTLV-1 and 2 IgG1 antibodies, serum/plasma samples are thawed, diluted in PBS (0.15M pH 7.2, 8.0 g/L NaCl , 2.0 g/L of KCl, 2.0 g/L of KH_2_PO_4_ and 1.15 g/L of Na_2_HPO_4_) supplemented with 3% FBS, centrifuged at 4 ºC, 14,000 rpm for 5 min and the supernatants are stored in the refrigerator until use. In 96-well plates with a U-shaped bottom, 50 µL of serum/plasma diluted in PBS-3% FBS and 50 µL of the previously fixed cell suspension (1 x 10^6^ cells/mL – MT-2 and MoT) are added. The mixture should be incubated at 37 °C for 30 min, washed with 150 µL of PBS-3% FBS by centrifugation (4 °C, 1,300 rpm for 10 min) and the supernatant discarded. To test for IgG1 antibodies, 50 µL of biotinylated anti-IgG1 antibody diluted in PBS-3% FBS and 10 µL of SAPE diluted in PBS-2% bovine serum albumin is added to each well of the plate. The mixture should be incubated at 37 °C for 30 min. After incubation, the cells should be washed once, as described above, and the supernatant discarded. Samples must be kept at 4 °C and protected from light until reading on a FACScalibur-BD flow cytometer. The maximum time for data collection must always be less than 24 hours. For each assay, a reaction control should be used, in this case, a control with secondary antibody.
