# Supplementary material for: Performance of immunological assays for universal and differential diagnosis of HTLV-1/2 infection in candidates for blood donations from the Brazilian Amazon
Source: PLoS One. 2024 Jul 5;19(7):e0298710. doi: 10.1371/journal.pone.0298710 (PMC11226060; doi:10.1371/journal.pone.0298710)
Supplement: S1 Table — (DOCX) [file pone.0298710.s002.docx]

Supplementary Table 1. Demographical and laboratorial records of candidates for blood donations with positive results for HTLV-1/2 after serological retesting using CLIA.

| **Variables** |  | **CLIA^*^ retesting of candidates for blood donations** |
| --- | --- | --- |
|  |  | **(n = 151)** |
| **Age**, years (median [IQR]) |  | 34 [18-61] |
| **Gender** | | |
| Male, n (%) |  | 69 (46%) |
| Female, n (%) |  | 82 (54%) |
| **CLIA^*^ Results** | | |
| Positive, n (%) |  | 93 (62%) |
| Negative, n (%) |  | 58 (38%) |
| RLU Positive, (median [IQR]) |  | 2.5 [1.3-81.4] |
| RLU Negative, (median [IQR]) |  | 0.1 [0.1-0.5] |
| **WB Results** |  |  |
| Positive, n (%) |  | 30 (20%) |
| Negative, n (%) |  | 111 (73%) |
| Indeterminate, n (%) |  | 10 (7%) |
| **FC-Simplex Results** | | |
| Positive, n (%) |  | 26 (17%) |
| Negative, n (%) |  | 125 (83%) |
| Positive PPFC, (median [IQR]) |  | 34.8 [28.3-48.1] |
| Negative PPFC, (median [IQR]) |  | 8.5 [6.6-11.8] |

*^*^CLIA: chemiluminescence assay; RLU: relative light units; WB: western blot; PPFC:* *percentage of positive fluorescent cells.*
