# Supplementary material for: Performance of immunological assays for universal and differential diagnosis of HTLV-1/2 infection in candidates for blood donations from the Brazilian Amazon
Source: PLoS One. 2024 Jul 5;19(7):e0298710. doi: 10.1371/journal.pone.0298710 (PMC11226060; doi:10.1371/journal.pone.0298710)
Supplement: S2 Table — (DOCX) [file pone.0298710.s003.docx]

Supplementary Table 2: Diagnostic Performance of Immunological Assays for the Detection of HTLV-1 and HTLV-2 Infection

|  | Accuracy  % (CI 95%) | Sensitivity  % (CI 95%) | Specificity  % (CI 95%) | PPV^#^  % (CI95) | NPV  % (CI95) |
| --- | --- | --- | --- | --- | --- |
| WB^*^ vs CLIA (N=141) | 62.4  (54.2-70.0) | 100  (88.7-100) | 52.3  (43.0-61.3) | 36.1  (26.6-46.9) | 100  (93.8-100) |
| WB vs FC-Simplex (N=141) | 91.5  (85.7-95.1) | 73.3  (55.6-85.8) | 96.4  (91.1-98.6) | 84.6  (66.5-93.9) | 93.0  (86.8-96.4) |
| CLIA^*^ vs FC-Simplex (n=151) | 55.6  (47.7-63.3) | 28.3  (20.1-38.) | 98.3  (91.0-99.7) | 96.3  (81.7-99.3) | 46.8  (38.2-55.5) |

**WB: Western Blot; CLIA: Chemiluminescence; FC-Simplex: FC-Simplex IgG1 (HTLV);* ^#^PPV: positive predictive value; NPV: negative predictive value.
