# Supplementary material for: Performance of immunological assays for universal and differential diagnosis of HTLV-1/2 infection in candidates for blood donations from the Brazilian Amazon
Source: PLoS One. 2024 Jul 5;19(7):e0298710. doi: 10.1371/journal.pone.0298710 (PMC11226060; doi:10.1371/journal.pone.0298710)
Supplement: S2 Fig — a) Anti- MT-2 (1:32) and anti-MoT (1:2,048) reactivity used to define the HTLV-1 and HTLV-2 diagnosis according to criterion 1, previously proposed by Pimenta de Paiva et al. [28]. b) Agreement between FC-Duplex HTLV-1/2 IgG1 and western blot results. PPFC: percentage of positive fluorescent cells; WB: western blot assay. (PDF) [file pone.0298710.s005.pdf]

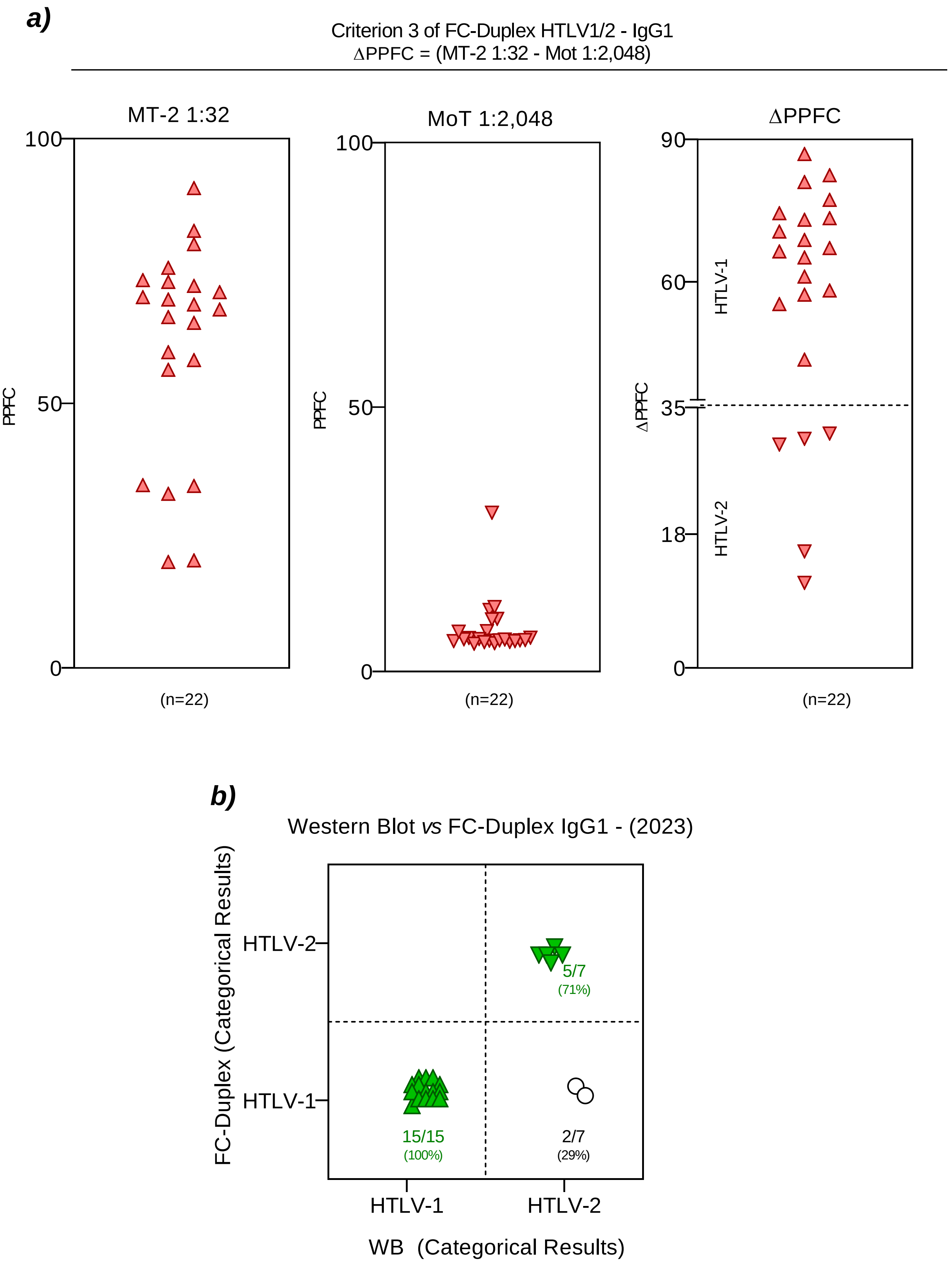

Supplementary Figure 2. Differential diagnosis of HTLV-1/2 infection after retesting using the FC-Duplex IgG1. ***a)*** Anti- MT-2 (1:32) and anti-MoT (1:2,048) reactivity used to define the HTLV-1 and HTLV-2 diagnosis according to criterion 1, previously proposed by Pimenta de Paiva et al. [25]. ***b)*** Agreement between FC-Duplex HTLV-1/2 IgG1 and western blot results. *PPFC: percentage of positive fluorescent cells; WB: western blot assay*.
